# Novel Biomarkers to Guide Immunotherapy De-Escalation in the Neoadjuvant Setting in Triple-Negative Breast Cancer

**DOI:** 10.3390/jpm13091313

**Published:** 2023-08-27

**Authors:** Shipra Gandhi

**Affiliations:** Department of Medicine, Roswell Park Comprehensive Cancer Center, Buffalo, NY 14263, USA; shipra.gandhi@roswellpark.org

Triple-negative breast cancer (TNBC) has the highest incidence of disease recurrence and distant metastases among breast cancer subtypes, leading to significant rates of morbidity and mortality. Neoadjuvant treatment with anthracycline- and taxane-based chemotherapy (AC-T) was the standard of care for early-stage TNBC, but pathological complete response (pCR) rates remained low, lingering at 25–35% [1,2,3]. With the recent FDA approval adding pembrolizumab, an immune checkpoint inhibitor, to an intense four-drug neoadjuvant chemotherapy (NAC) backbone of anthracycline–cyclophosphamide with carboplatin–paclitaxel (KEYNOTE-522) [4], the pCR rate has increased to 65%. However, this advantage has come at the cost of serious immune-related adverse events (irAEs), such as adrenal insufficiency, hypophysitis, colitis, hepatitis, pancreatitis, myocarditis, myositis, type 1 diabetes mellitus, and encephalitis, which can be permanent, life-threatening and disabling. Almost 13% of patients in the pembrolizumab-NAC group in KEYNOTE-522 experienced grade 3–5 irAEs (with 0.3% deaths from irAEs), versus only 1.0% in the group with NAC alone [4].

It is increasingly clear that about 30–50% of patients can attain a pCR with NAC alone, with neoadjuvant checkpoint inhibition only helping another 10–15% patients to attain pCR. Due to the absence of biomarkers capable of predicting who these 10–15% patients could be, the current clinical practice is to give immune checkpoint inhibitors to all patients, resulting in the overtreatment of a significant number of patients. Therefore, it is crucial that the need for immune checkpoint inhibitors in the neoadjuvant setting be carefully evaluated. However, currently, there is a lack of biomarkers that could help to select these TNBC patients and there is a need for every effort to be dedicated to identifying responders to immune checkpoint inhibitors upfront.

Although PDL1 is an established predictive biomarker in the advanced setting, in KEYNOTE 522, PDL1 did not differentiate responders from non-responders in the early setting, as both PDL1-positive and PDL1-negative patients derived benefit from the addition of neoadjuvant pembrolizumab [5]. The NeoTRIP study was a clinical trial where TNBC patients were enrolled and randomized to neoadjuvant carboplatin and nab-paclitaxel with or without atezolizumab. This was followed by surgery and then four cycles of an adjuvant anthracycline regimen. The study showed that the addition of atezolizumab did not significantly increase the rate of pCR in women with TNBC. Interestingly, as opposed to KEYNOTE 522, the presence of PDL1 expression was the most significant factor influencing the rate of pCR (OR = 2.08, *p* < 0.0001). One possible hypothesis for the different predictive ability of PDL1 could be that, in the case of KEYNOTE 522, anthracyclines induced immunogenic cell death that could have increased the likelihood of response of PDL1-negative tumors where immune priming phase is dysfunctional [6].

In GeparNuevo study, TNBC patients were randomized to receive neoadjuvant nab-paclitaxel followed by doxorubicin–cyclophosphamide with or without durvalumab. Interestingly, this study showed that, in the case of high-TMB tumors, the distant disease-free survival was similar between both arms of the trial (durvalumab vs. placebo: HR 0.95 (95% CI, 0.19–4.69), *p* = 0.95). However, within the low-TMB group, there was a better disease-free survival in the durvalumab–chemotherapy combination arm, in contrast to the arm treated only with chemotherapy [7].

The NeoPACT trial enrolled patients with stage I–III TNBC who received a carboplatin–docetaxel backbone with pembrolizumab in the neoadjuvant setting. Conversely, in Arm B of the NeoSTOP trial, patients were treated with neoadjuvant docetaxel-carboplatin combination alone. The authors investigated a 14-gene IGG immune signature [8]. In the NeoPACT trial, the 14-gene IGG signature was significantly associated with improved pCR (OR = 1.105, 95% CI 1.019–1.197). The pCR rates in IGG-high (≥median) and IGG-low (≤median) groups were 71% and 44%, respectively (OR = 3.15, 95% CI 1.42–6.99). Among the patients treated on NeoSTOP who received neoadjuvant chemotherapy, there was no association of IGG signature with pCR. The authors concluded that a high expression of this IGG immune signature in baseline pretreatment tumor samples in early-stage TNBC could be significantly associated with pCR following pembrolizumab-based neoadjuvant chemotherapy and may help to select patients who would selectively benefit from this approach. The same group also investigated the impact of proliferation gene expression on efficacy of neoadjuvant systemic therapy in sTIL-high (≥20% sTILs) and sTIL-low (<20% sTILs) TNBC [9]. ImSig was a proliferation signature score (ProlifSig computed from RNA sequencing data and samples were classified as ProlifSig-high (≥median) or ProlifSig-low (<median). These ProlifSig values were determined for the prediction of pCR in sTIL-high and sTIL-low groups. In the sTIL-high group, ProlifSig was not associated with pCR, either as a continuous score or when assessed as high/low categories. However, in the sTIL-low group, ProlifSig was significantly associated with pCR when assessed as high/low categories (pCR 75% vs. 29% in ProlifSig-high and ProlifSig-low groups, respectively, OR = 3.18, 95% CI = 1.03–9.86). Therefore, the ProlifSig could identify a subgroup of low-immunity TNBCs capable of achieving substantial rates of pCR with neoadjuvant chemoimmunotherapy.

The ability of MHC-II expression on tumor cells to predict immunotherapy-specific benefits in the neoadjuvant breast cancer setting as a potential biomarker was assessed. This study utilized patients who were treated with (i) durvalumab and neoadjuvant chemotherapy, (ii) pembrolizumab and neoadjuvant chemotherapy and (iii) chemotherapy alone. The study showed that the quantitative assessment of MHC-II on the tumor cells was predictive of benefit from durvalumab and neoadjuvant chemotherapy, and pembrolizumab and neoadjuvant chemotherapy, but not neoadjuvant chemotherapy alone [10].

More fascinating research from NeoTRIP showed that cell–cell interactions also have predictive value in determining responses. A high density of antigen-presenting cells with high expression levels of PDL1 and the immunosuppressive molecule IDO, as well as a high density of of epithelial cells with high expression levels of the CD56 neuroendocrine marker, was associated with higher pCR rate in patients who received atezolizumab plus chemotherapy, but not in patients who only received chemotherapy. In addition, there was also a high degree of spatial connectivity between epithelial cells and specific tumor microenvironment cells—for instance, CD8+ T cells with granzyme B or PD-1 expression correlated with a significant increase in the pCR rate after atezolizumab administration, whereas a lower expression of these markers was associated with similar pCR rates between the atezolizumab arm and the chemotherapy-alone arm [11].

Although exploratory analyses of biomarkers from these clinical trials are intriguing, at this time, the standard of care for early-stage high-risk TNBC patients continues to be neoadjuvant immune checkpoint inhibition with chemotherapy. Further validation of these biomarkers is needed in future clinical trials to help the scientific community to identify the patients where neoadjuvant chemotherapy alone will be sufficient and the use of immune checkpoint inhibitors can be safely spared.
